# BENviewer: a gene interaction network visualization server based on graph embedding model

**DOI:** 10.1093/database/baab033

**Published:** 2021-05-28

**Authors:** Yunqing Liu, Yunchi Zhu, Chunpeng He, Zuhong Lu

**Affiliations:** State Key Laboratory of Bioelectronics, Southeast University, Sipailou No.2, Nanjing, Jiangsu 210096, China; State Key Laboratory of Bioelectronics, Southeast University, Sipailou No.2, Nanjing, Jiangsu 210096, China; State Key Laboratory of Bioelectronics, Southeast University, Sipailou No.2, Nanjing, Jiangsu 210096, China; State Key Laboratory of Bioelectronics, Southeast University, Sipailou No.2, Nanjing, Jiangsu 210096, China

## Abstract

BENviewer is a brand-new online gene interaction network visualization server based on graph embedding models. With mature graph embedding algorithms applied on several interaction network databases, it provides human-friendly 2D visualization based on more than 2000 biological pathways, and these results present not only genes involved but also tightness of interactions in an intuitive way. As a unique visualization server introducing graph embedding application for the first time, it is expected to help researchers gain deeper insights into biological networks beyond generating results explainable by existing knowledge. Additionally, operation flow for users is simplified to greater extent in its current version; meanwhile URL optimization contributes to data acquisition in batch for further analysis. BENviewer is freely available at http://www.bmeonline.cn/BENviewer, besides it is open-sourced at https://github.com/SKLB-lab/BENviewer, http://benviewer.bmeonline.cn.

## Introduction

In view of the important role of molecular interaction network in life activities, bioinformatics researchers have developed various network visualization tools. For example, Cytoscape (1) presents biological entity interaction as a network topographic structure, which helps researchers intuitively observe gene interaction; SpringScape (2) interprets microarray data by dynamically combining related data and focusing on a particular feature; Goel’s method (3) is aimed at visualizing and analyzing protein–protein interaction networks in 4D space; BioLayout Express^3D^ (4) is designed for visualization and analysis of graphs derived from biological data.

Although the above tools facilitate data-driven biological network research to some extent, they may fail to help researchers gain deeper insight into the knowledge behind these biological entities. For visualization, it is expected that genes with more interactions locate closer to each other and form a cluster, which indicates their participation in related biological process. Gene Set Enrichment Analysis (GSEA) (5) may achieve that goal by analyzing single predefined functional group, but it has difficulty presenting the whole gene distribution in an organism. Time-consuming hypothesis testing process and crosstalking between pathways also exert negative effects on GSEA approach.

Here we introduce BENviewer, a gene interaction network visualization server based on graph embedding model (6). Network embedding visualization has obtained several successful applications during long time development (6), for example, in natural language processing, Word2vec transforms word into a real vector to characterize the similarity between words by cluster distance, offering researchers capability of browsing the whole vocabulary distribution in the visual space for their interested information. Palash *et al* (6). summarized graph embedding applications in biomedical networks such as link prediction and node classification, where dimensionality reduction is always necessary. Although a part of similar work on biomedical networks (7), there seem to be few reports about graph embedding application in biological interaction network visualization. BENviewer is developed to provide a new approach for gene interaction network visualization. BENviewer is expected to simply series of time-consuming manual visualization operations to one semi-automatic process; meanwhile its demonstration will be more user-friendly.

## Materials and methods

### Workflow overview

**Figure 1.**
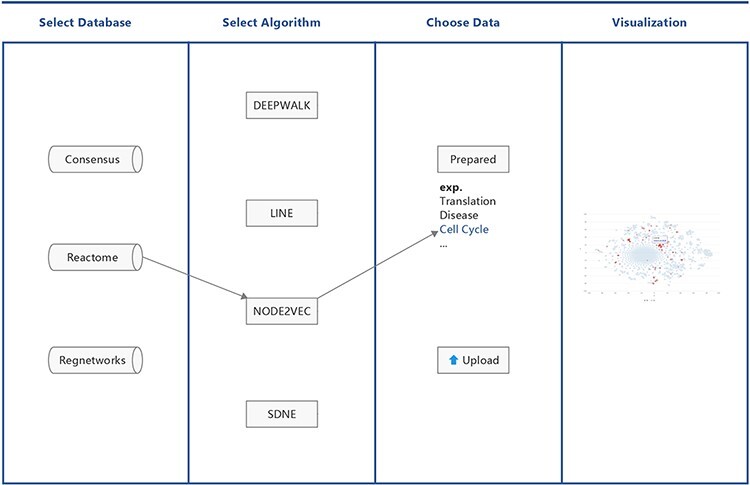
Workflow of BENviewer.

BENviewer is based on three pathway interaction databases: ConsensusPathDB (8), Reactome (9) and RegNetwork (10). Four graph embedding algorithms, Deepwalk (11), LINE (12), Node2vec (13) and SDNE (14), are employed by these databases to reduce the high-dimensional interaction network to 2D. Hence, in total, 12 datasets are generated. After choosing one dataset by selecting its corresponding database and algorithm in turn, users can either pick out prepared gene lists related to biological functions and pathways or upload their own gene lists of interest for visualization. Results are provided in the form of a scatterplot, where users’ genes of interest are highlighted in red (Figure 1).

### Implementation details

#### Technology stack

BENviewer is developed under the Linux, Nginx, MySQL and PHP (LEMP) stack. The Highcharts library was imported for scatterplotting, while all other web functions were implemented by PHP 7.2. Nginx works as web server.

The web server has been open-sourced under MIT license (https://github.com/SKLB-lab/BENviewer/blob/master/LICENSE), and most services have been built into Docker images—beneficial for cross-platform deployment and extended development.

Prepared gene lists are stored in three MySQL databases named ‘BEN.consensus’, ‘BEN.reactome’ and ‘BEN.regnetwork’. Each database consists of four tables corresponding to four algorithms with structure as shown in Table 1.

**Table 1.** Structure of BEN database table

Name	Type	Description	Sample
gene	TEXT	Gene name	AIMP1
X	DOUBLE	Results of graph embedding models	7.91651
Y	DOUBLE		−9.38209
func	TEXT	Related biological function or pathway	REACTOME_TRANSLATION

#### Graph embedding models

The goal of the graph embedding algorithm is to find the low-dimensional vector representation of the node, which can reflect the network properties. Although different methods focus on different aspects of interaction network attributions, it is a consensus that nodes with similar structural roles in networks should be embedded closer.

Deepwalk is a novel approach for learning latent representations of vertices in a network. Inspired by Word2vec, its workflow is as follows: select a specific point as the starting point; do a random walk to get a sequence of points and treat the obtained sequence as a sentence and learn it with Word2vec to get the representation vector of the node. Deepwalk can obtain the local context information of a node in the graph by random walk, which means it focuses on first- and second-order proximities.

Similar to Deepwalk, Node2vec maintains the high-order proximity between nodes by maximizing the probability of nodes in the sequence obtained by random walk. Their most significant difference is that Node2vec utilizes a biased random walk to trade-off between local and global views of the network. This method balances proximity and structure for higher quality and more informative embedding.

LINE initially constructs an objective function with existing edges in the graph, which explicitly depicts the first-order and second-order neighbor relations. Then, the expression vector of the node is learned through the optimization method.

SDNE was published together with Node2vec in the 2016 Knowledge Discovery in Data (KDD) conference. It is regarded as the first attempt to apply deep learning method in Network Representation Learning (NRL). In contrast to LINE, SDNE adopts an auto-encoder to optimize the first-order and second-order proximities at the same time. Its learned vector representation can retain the local and global structures, remaining robust to sparse networks.

These algorithms were employed by three interaction databases with all pathways formatted into edge lists. Among them, Reactome consists of all entity interactions in human biological pathways, RegNetwork focuses on the transcription factor or microRNA regulating target gene set and ConsensusPathDB integrates interaction networks including binary and complex protein–protein, genetic, metabolic, signaling, gene regulatory and drug–target interactions. After embedding each node into a real vector with no matter which model above, t-SNE (15) or UMAP (16) was selected based on the actual effect to reduce the dimension to 2D for human-friendly and intuitive visualization. All the results are stored in MySQL databases mentioned above.

### Input and output

**Figure 2.**
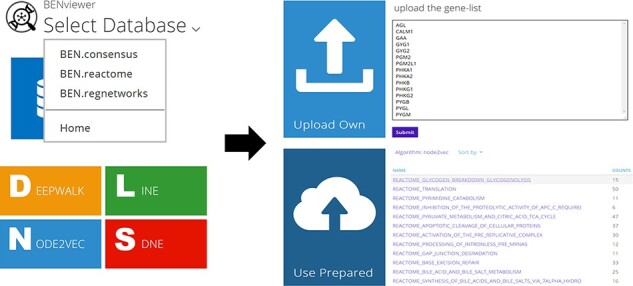
Operation process and related pages.

**Figure 3.**
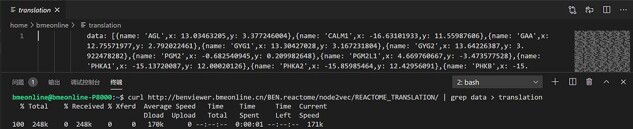
One approach using Linux commands to acquire the raw data.

Users are allowed to select BEN-database and algorithm directly by URL in the format of ‘<domain of BENviewer>/<db>/<algo>/’ besides clicking corresponding buttons on page (Figure 2). Visualization of preset gene lists requires no inputs. It is only necessary for users to choose their interested item in the list provided by server. If they tend to upload their own gene list, an input box will be displayed on the page. The input is limited to gene symbols searchable in National Center for Biotechnology Information (NCBI), while genes without ENTREZ ID will not be processed. As a URL optimization, to enable faster access, the name of selected biological function or pathway will be appended to the end of current URL (Figure 3; Supplementary Material 1).

**Figure 4.**
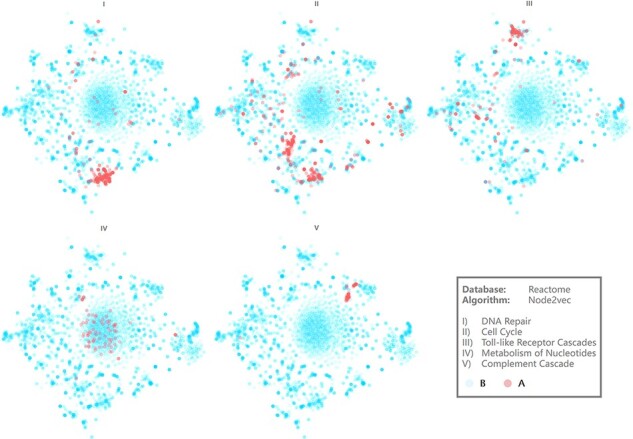
Visualization results of several pathways. Axis is hidden for more intuitive demonstration.

The output is a 100% × 100% 2D scatterplot (Figure 1 right; Figure 4). Genes are classified into Group A and Group B, where Group A genes displayed as red dots come from user’s selected or uploaded list. A click on the legend location of one group will hide all the group’s genes on the plot, and a hover on one gene will trigger a floating window demonstrating its name and link to NCBI Gene (Figure 1 right), which records its detailed information. Dot color and transparency can be adjusted by adding RGB at the end of current URL, for example, adding ‘138,43,226,0.4&0,255,0,.1’ will change dot colors to green and purple.

The raw data of output scatterplot is accessible in the source code of the result page, which is not only convenient for direct downloading but also conducive for API development with multiple computer languages (Figure 3).

## Results

Up to now, there are totally 2109 biological pathways in BEN databases. The quantity of included genes is as follows: 19 286 in ‘BEN.consensus’, 5444 in ‘BEN.reactome’ and 23 332 in ‘BEN.regnetwork’. UMAP is chosen as the dimensionality reduction method for results generated by SDNE, while all other data are processed by t-SNE (Supplementary Material 2 Figure 1 for details).

Figure 4 demonstrates the visualization results of five representative biological pathways on BENviewer: DNA repair, cell cycle, toll-like receptor cascades, metabolism of nucleotides and complement cascade (Supplementary Material 1 for raw pages with URLs). They are numbered Pathways I–V for short in the following.

Interactions in Pathway IV seem to be relevantly weaker in view of clusters on scatter plot appear to be loose relevantly. On the contrary, interactions in other pathways are observed as tight and centralized, among which Pathway V emerges to be most obvious. Although the reason for this phenomenon remains to be analyzed in detail, it is undeniable that comparison of such visualization results gives researchers capability to recognize the tightness difference between biological function networks. Network structural characteristics may explain this difference theoretically because nodes with more analogous neighbor relations will be clustered more tightly by graph embedding model, indicating the similarity of their roles in regulation pathways. Enrichment degree of biological function can also be revealed by the tightness difference in a more intuitive way. For example, result from Gene Ontology (GO) enrichment analysis conducted on A group genes in Pathways IV and V was that GO terms involving Pathway V presented to be more concentrated and evenly distributed in view of molecular function (Supplementary Material 2 Tables 1 and 2 for details).

Two gene clusters appear in visualization result of Pathway II, which can be explained as some of these genes mainly contribute to mitosis while the others involve in aborting cell circle at checkpoints (Supplementary Material 2 Figure 2 for more evidence). Pathway I’s clustering center has high coincidence with one of those two, in line with the biological knowledge that most DNA repairing processes happen at synthesis in cell cycle.

**Figure 5.**
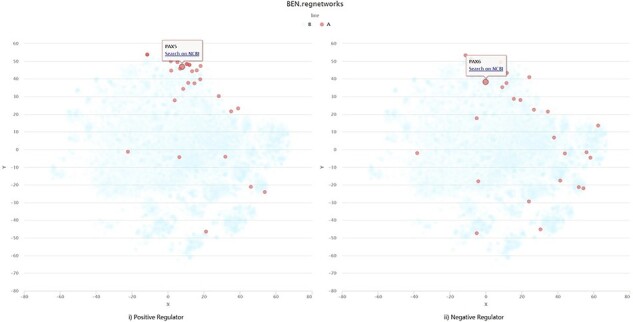
Visualization example of self-uploading data.

There is also an example for self-uploading data visualization (Supplementary Material 1 for raw pages with URLs). The gene lists illustrated in Figure 5 comes from Corces *et al.*’s work (17), among which genes exerting positive regulation on motif appear to interact much more closely than the negative regulators. Such visualization result as a macro-overview for regulome dynamics is basically consistent with the results of their work.

## Discussion

For one thing, it is commonly accepted that results from excellent visualization tools not only conform to existing knowledge but also assist people in discovering anything brand-new or unknown in time. According to the above description, BENviewer seems to perform well enough from this perspective. For another thing, it is also required to be easily accessible and user-friendly as a web server. This aspect is expected to be gradually improved along with the increasing number of visits and feedbacks from users worldwide.

In future versions of BENviewer, emerging biological interaction network databases will be transformed and added into BEN datasets in time, with more algorithms applied, which are not necessarily limited to graph embedding models. As an extension, APIs for visualization result acquisition as well as analysis tools based on them are under development. We hope that BENviewer is conducive to the virtuous 3A [Acquisition-Analysis-Application (18)] circle in both biology research and algorithm development.

## Supplementary Material

baab033_Supp

## Data Availability

The web server is available at http://www.bmeonline.cn/BENviewer. This website is free and open to all users with no login required. The source codes are open at https://github.com/SKLB-lab/BENviewer. Forking, issues and pull requests are always welcome. Data generated by t-SNE and UMAP are both provided for customized deployment.
