# Supplementary material for: BENviewer: a gene interaction network visualization server based on graph embedding model
Source: Database (Oxford). 2021 May 28;2021:baab033. doi: 10.1093/database/baab033 (PMC8163240; doi:10.1093/database/baab033)

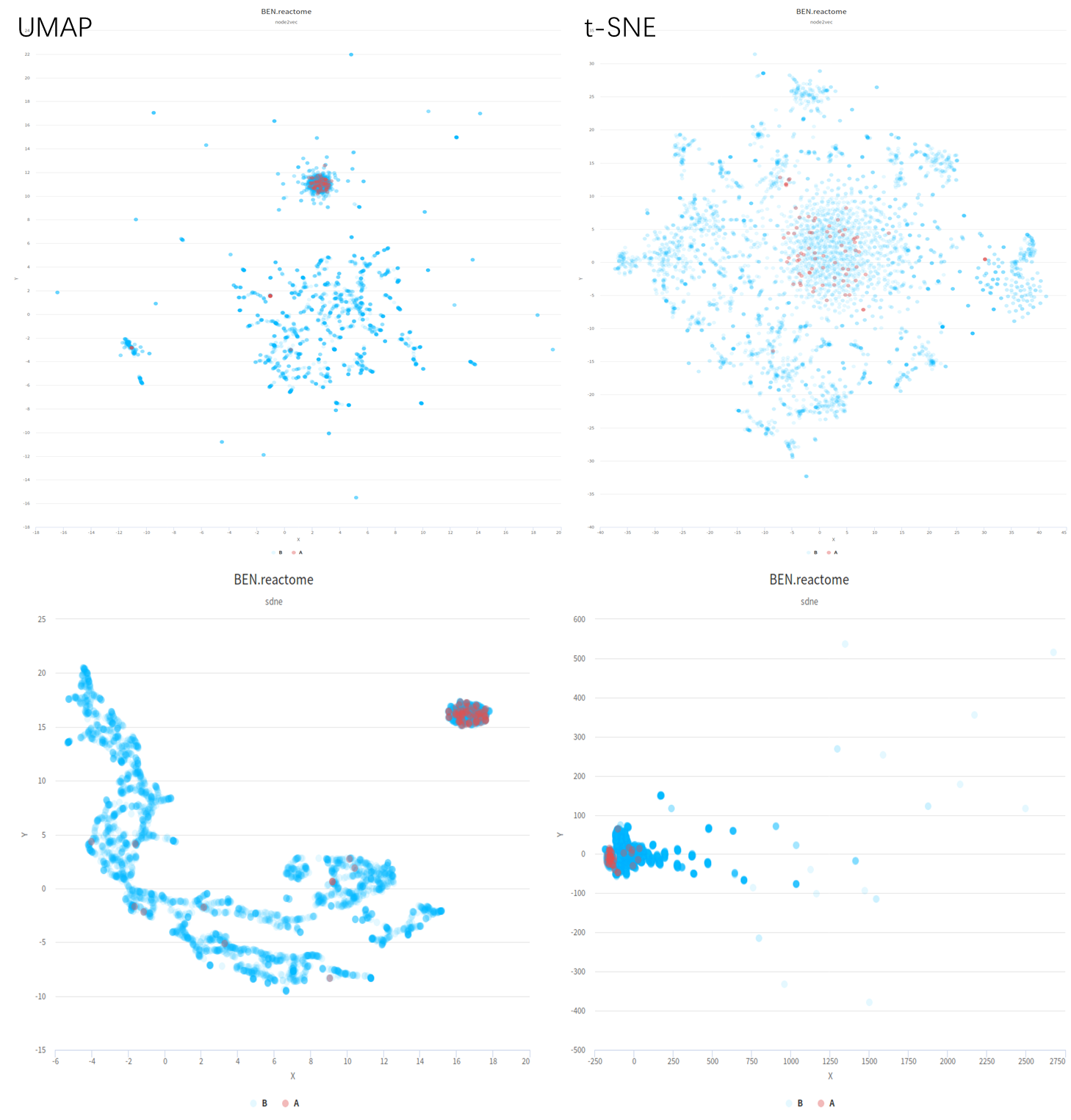


Fig 1 Example for visualization comparison between UMAP and t-SNE on Pathway IV. Although UMAP performs better than t-SNE on all 12 BEN datasets according to clustering evaluation indicators such as *Calinski-Harabasz Score* [1] and *Silhouette Coefficient* [2] under most parameters, it’s common that results from UMAP turn out to be too dense in the visual space on the default graphic attributes (color A: 223 83 83 .4; color B: 0 191 255 .1), which not only weakens the tightness difference but also brings out strong background noise visually. It is concluded that excellent clustering effect is not equivalent to excellent visualization effect. Yet UMAP is chosen as the dimensionality reduction method for results generated by SDNE for its obvious advantages against t-SNE both on clustering evaluation indicators and actual visualization effect.

[1] Cengizler, Caglar, and M. Kerem Un. "Evaluation of Calinski-Harabasz Criterion as Fitness Measure for Genetic Algorithm Based Segmentation of Cervical Cell Nuclei." Journal of Advances in Mathematics and Computer Science (2017): 1-13.

[2] Aranganayagi, S., and Kȷ Thangavel. "Clustering categorical data using silhouette coefficient as a relocating measure." International conference on computational intelligence and multimedia applications (ICCIMA 2007). Vol. 2. IEEE, 2007.


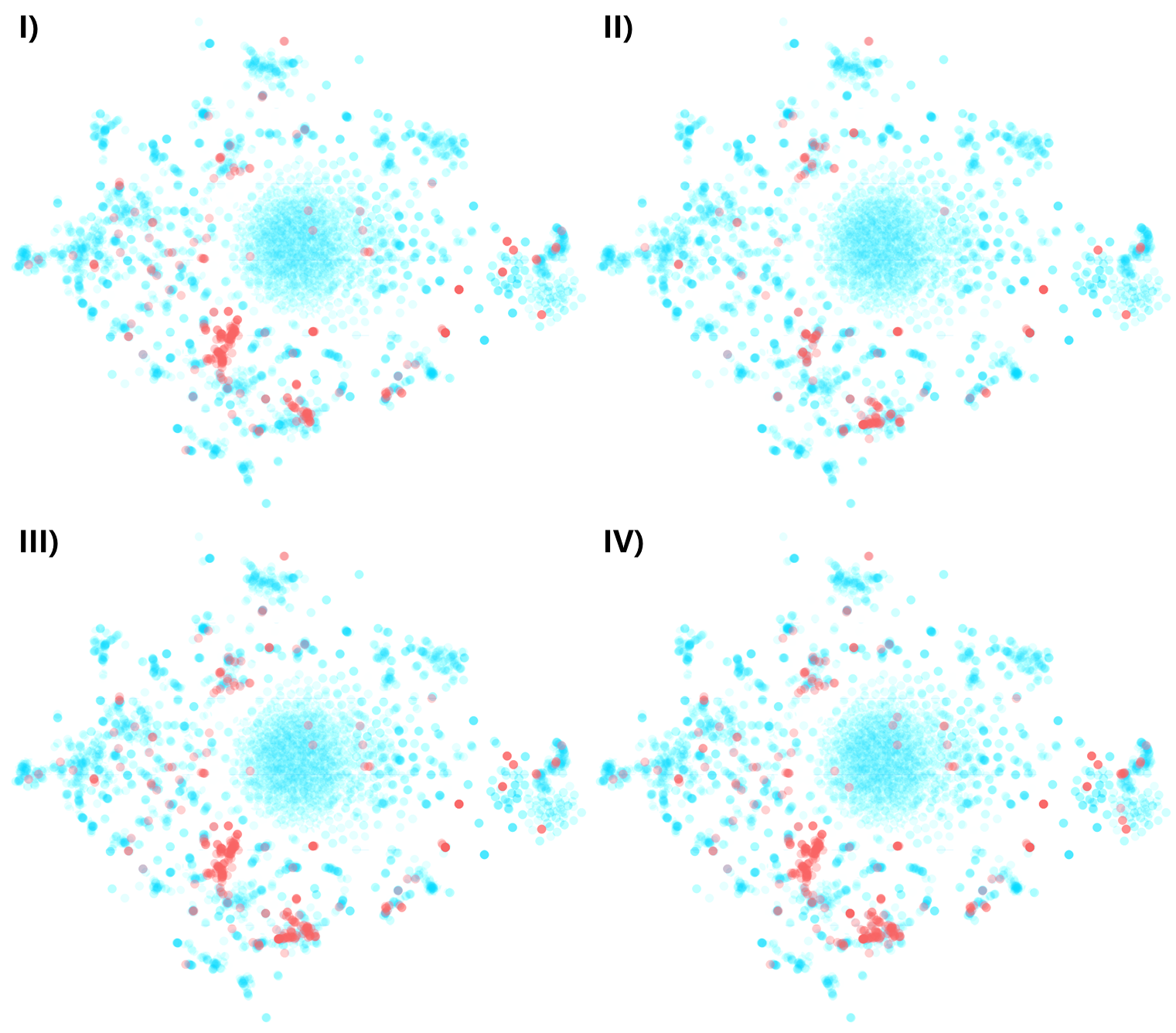


Fig 2 The intersection of CELL_CYCLE_MITOTIC and CELL_CYCLE_CHECKPOINTS is significantly similar to CELL_CYCLE. These two almost constitute a complement.

I) REACTOME_CELL_CYCLE_MITOTIC

II) REACTOME_CELL_CYCLE_CHECKPOINTS

III) The intersection of CELL_CYCLE_MITOTIC and CELL_CYCLE_CHECKPOINTS

IV) REACTOME_CELL_CYCLE

Table 1 GO enrichment of Pathway IV. 28 GO terms were enriched totally, most among which turned out to be enzyme activity related to nucleotide metabolism, such as nucleobase-containing compound kinase, nucleoside diphosphate kinase, phosphotransferase, etc. Both origins and functions of these enzymes present to be relatively diverse, explaining for this pathway’s weaker tightness to some extents.

| **ID** | **GeneRatio** | **BgRatio** | **pvalue** | **p.adjust** | **qvalue** |
| --- | --- | --- | --- | --- | --- |
| **GO:0019205** | 21/77 | 43/18352 | 5.91E-40 | 6.98E-38 | 3.92E-38 |
| **GO:0004550** | 12/77 | 18/18352 | 2.2E-25 | 1.3E-23 | 7.31E-24 |
| **GO:0016776** | 13/77 | 40/18352 | 4.73E-22 | 1.86E-20 | 1.05E-20 |
| **GO:0017110** | 9/77 | 11/18352 | 1.36E-20 | 4E-19 | 2.25E-19 |
| **GO:0050145** | 9/77 | 21/18352 | 7.01E-17 | 1.66E-15 | 9.3E-16 |
| **GO:0008253** | 6/77 | 13/18352 | 7.5E-12 | 1.47E-10 | 8.29E-11 |
| **GO:0008252** | 6/77 | 15/18352 | 2.17E-11 | 3.66E-10 | 2.06E-10 |
| **GO:0047429** | 5/77 | 11/18352 | 5.16E-10 | 7.61E-09 | 4.28E-09 |
| **GO:0016763** | 7/77 | 50/18352 | 1.5E-09 | 1.96E-08 | 1.1E-08 |
| **GO:0016810** | 9/77 | 121/18352 | 1.93E-09 | 2.28E-08 | 1.28E-08 |
| **GO:0016814** | 6/77 | 35/18352 | 6.59E-09 | 7.07E-08 | 3.97E-08 |
| **GO:0016725** | 4/77 | 10/18352 | 5.9E-08 | 5.8E-07 | 3.26E-07 |
| **GO:0019239** | 5/77 | 32/18352 | 2.1E-07 | 1.91E-06 | 1.07E-06 |
| **GO:0000287** | 9/77 | 217/18352 | 3.08E-07 | 2.59E-06 | 1.46E-06 |
| **GO:0042578** | 11/77 | 369/18352 | 3.82E-07 | 3E-06 | 1.69E-06 |
| **GO:0016791** | 9/77 | 277/18352 | 2.35E-06 | 1.73E-05 | 9.74E-06 |
| **GO:0004551** | 3/77 | 19/18352 | 6.56E-05 | 0.000455 | 0.000256 |
| **GO:0008144** | 5/77 | 104/18352 | 7.58E-05 | 0.000497 | 0.000279 |
| **GO:0015035** | 3/77 | 22/18352 | 0.000103 | 0.000641 | 0.00036 |
| **GO:0016757** | 7/77 | 275/18352 | 0.000154 | 0.000911 | 0.000512 |
| **GO:0001882** | 8/77 | 389/18352 | 0.000223 | 0.001252 | 0.000704 |
| **GO:0030515** | 3/77 | 32/18352 | 0.000323 | 0.001731 | 0.000973 |
| **GO:0015036** | 3/77 | 41/18352 | 0.000675 | 0.003463 | 0.001946 |
| **GO:0004000** | 2/77 | 12/18352 | 0.001116 | 0.005268 | 0.00296 |
| **GO:0016668** | 2/77 | 12/18352 | 0.001116 | 0.005268 | 0.00296 |
| **GO:0015037** | 2/77 | 13/18352 | 0.001315 | 0.00597 | 0.003355 |
| **GO:0019215** | 2/77 | 15/18352 | 0.001761 | 0.007696 | 0.004325 |
| **GO:0016667** | 3/77 | 59/18352 | 0.00195 | 0.008218 | 0.004619 |


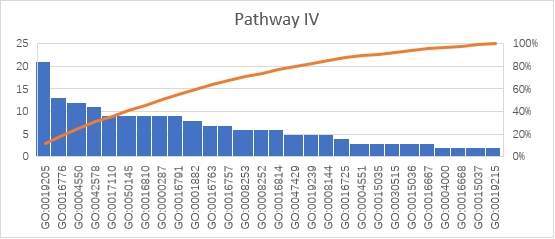


Table 2 GO enrichment of Pathway V. 21 GO terms were enriched totally, most of which represent molecular activity involving in complement cascade, such as complement binding, carbohydrate binding and exogenous protein binding. They present to be "receptor-centric" functionally, leading to stronger interaction tightness.

| **ID** | **GeneRatio** | **BgRatio** | **pvalue** | **p.adjust** | **qvalue** |
| --- | --- | --- | --- | --- | --- |
| **GO:0001848** | 8/46 | 21/18352 | 1.63E-16 | 1.33E-14 | 6.85E-15 |
| **GO:0004875** | 5/46 | 12/18352 | 6.18E-11 | 2.53E-09 | 1.3E-09 |
| **GO:0001618** | 5/46 | 74/18352 | 1.12E-06 | 2.3E-05 | 1.18E-05 |
| **GO:0140272** | 5/46 | 74/18352 | 1.12E-06 | 2.3E-05 | 1.18E-05 |
| **GO:0030246** | 7/46 | 267/18352 | 4.2E-06 | 6.89E-05 | 3.54E-05 |
| **GO:0001846** | 3/46 | 15/18352 | 6.57E-06 | 8.97E-05 | 4.61E-05 |
| **GO:0005537** | 3/46 | 20/18352 | 1.63E-05 | 0.000191 | 9.81E-05 |
| **GO:0140375** | 5/46 | 135/18352 | 2.15E-05 | 0.000221 | 0.000113 |
| **GO:0048306** | 4/46 | 85/18352 | 6.03E-05 | 0.000517 | 0.000265 |
| **GO:0004252** | 5/46 | 169/18352 | 6.3E-05 | 0.000517 | 0.000265 |
| **GO:0008236** | 5/46 | 187/18352 | 0.000102 | 0.000758 | 0.000389 |
| **GO:0017171** | 5/46 | 191/18352 | 0.000112 | 0.000768 | 0.000394 |
| **GO:0048029** | 3/46 | 69/18352 | 0.000688 | 0.004338 | 0.002228 |
| **GO:0004866** | 4/46 | 183/18352 | 0.001126 | 0.006484 | 0.003329 |
| **GO:0050750** | 2/46 | 21/18352 | 0.001252 | 0.006484 | 0.003329 |
| **GO:0030414** | 4/46 | 189/18352 | 0.001268 | 0.006484 | 0.003329 |
| **GO:0061135** | 4/46 | 192/18352 | 0.001344 | 0.006484 | 0.003329 |
| **GO:0030247** | 2/46 | 25/18352 | 0.001777 | 0.008097 | 0.004158 |
| **GO:0070325** | 2/46 | 26/18352 | 0.001922 | 0.008297 | 0.00426 |
| **GO:0004181** | 2/46 | 29/18352 | 0.00239 | 0.0098 | 0.005032 |
| **GO:0061134** | 4/46 | 229/18352 | 0.002556 | 0.009981 | 0.005125 |


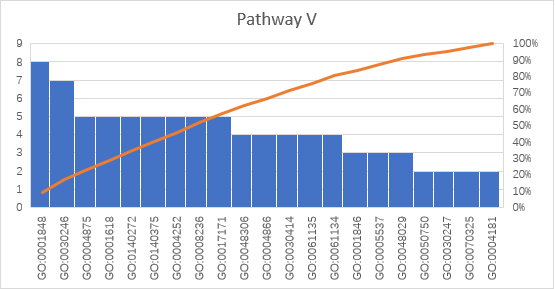

Supplement: baab033_Supp [file baab033_supp.zip › Supporting_Material_2_R2- clean.docx]
