# Supplementary figures and images for: BENviewer: a gene interaction network visualization server based on graph embedding model
Source: Database (Oxford). 2021 May 28;2021:baab033. doi: 10.1093/database/baab033 (PMC8163240; doi:10.1093/database/baab033)

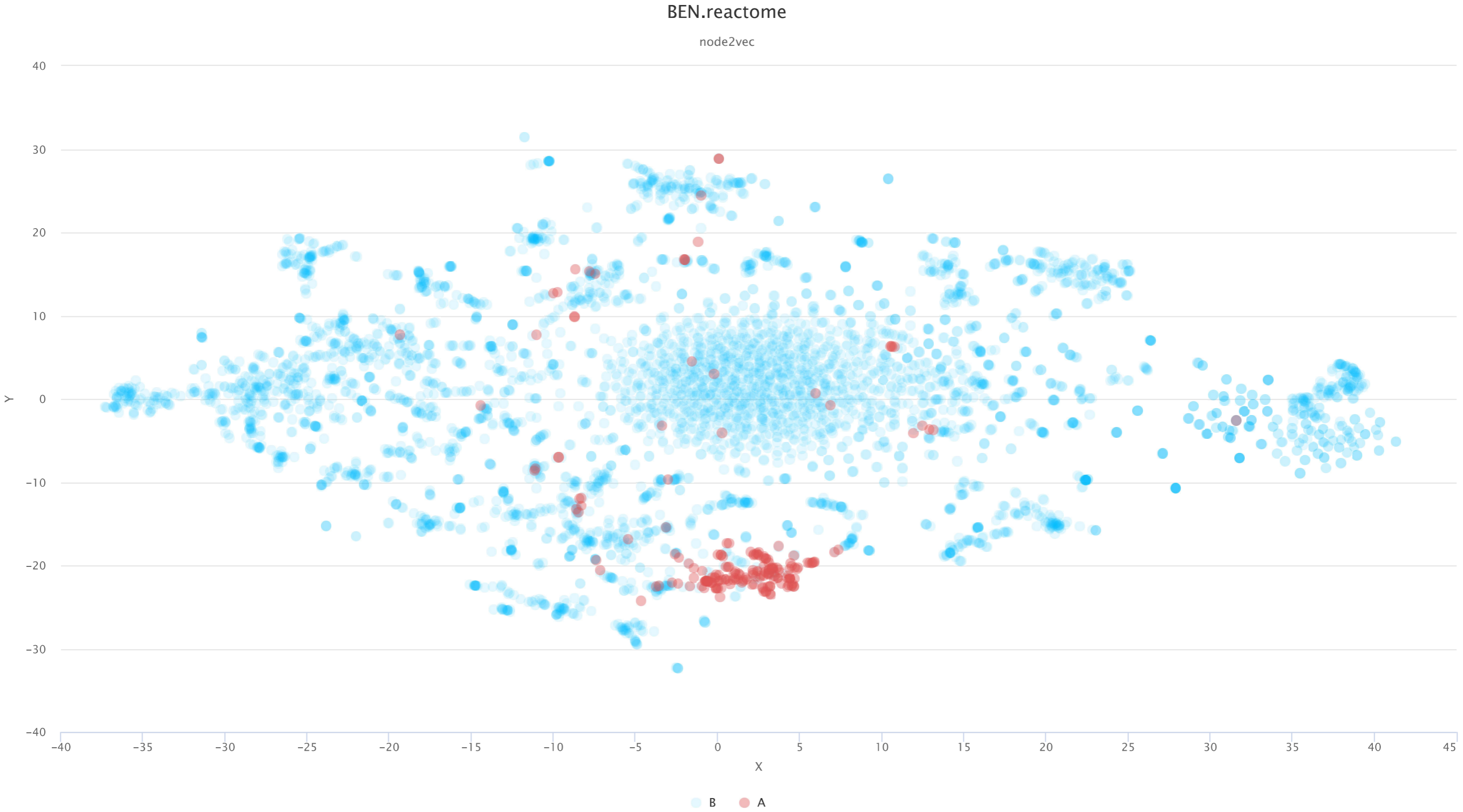

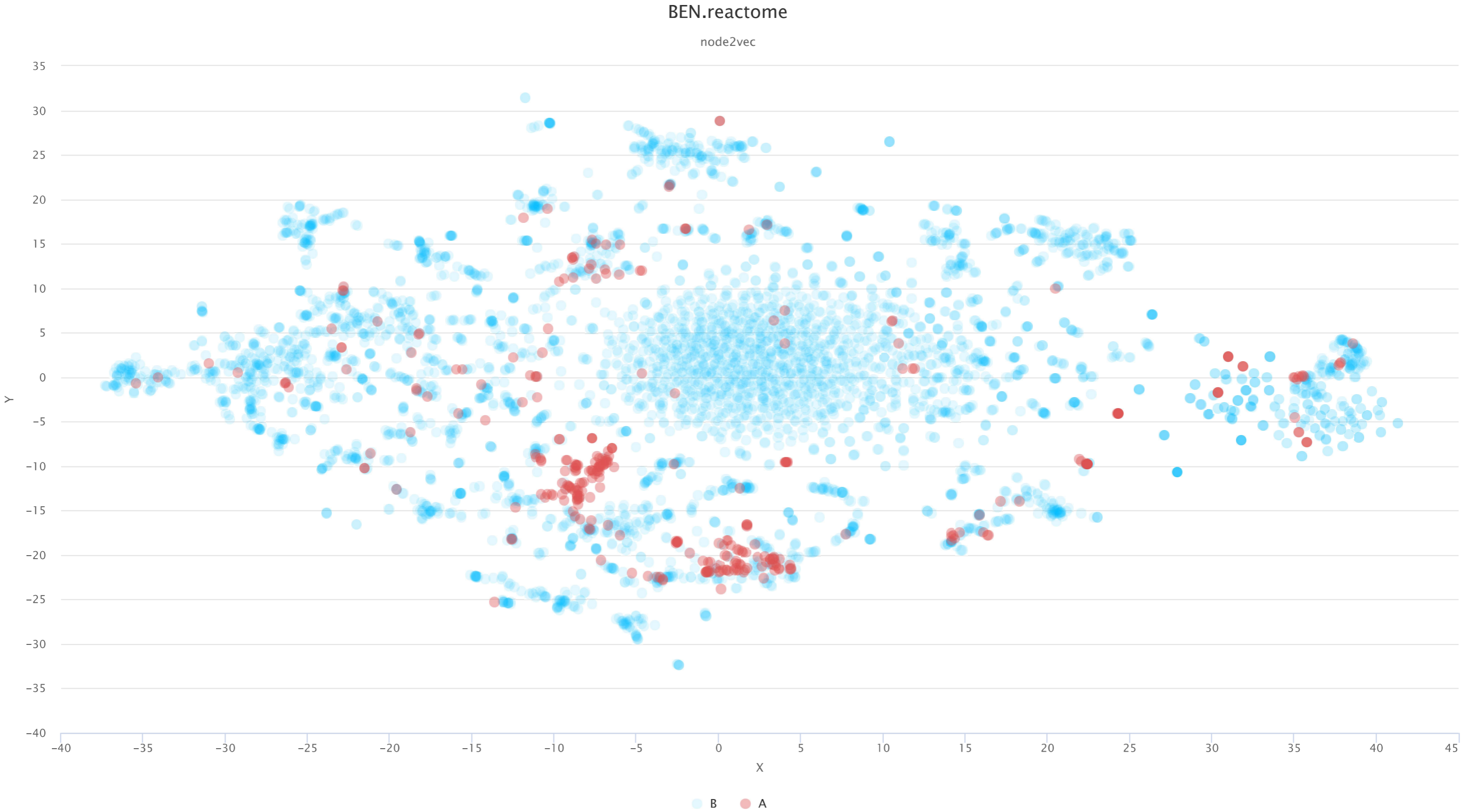

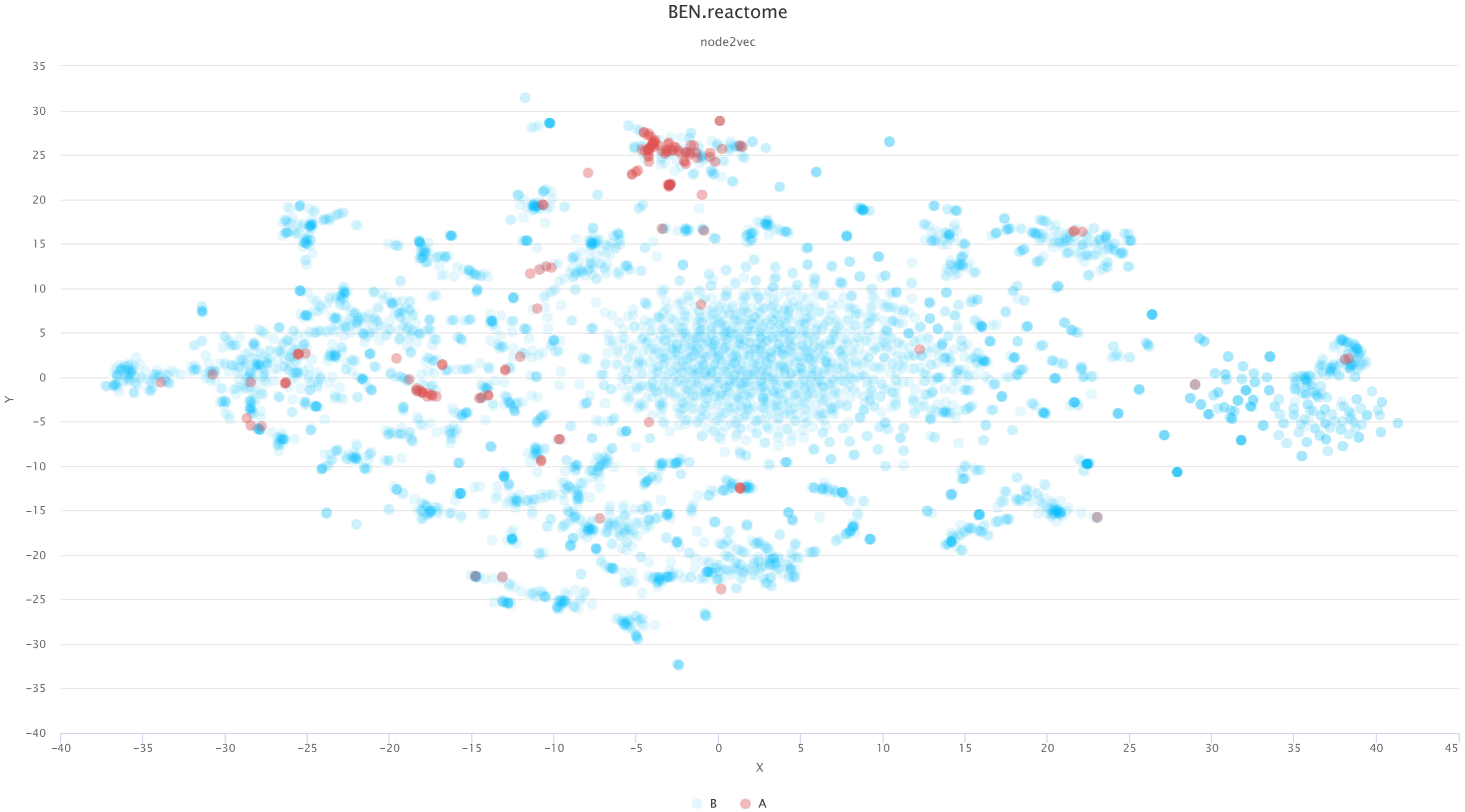

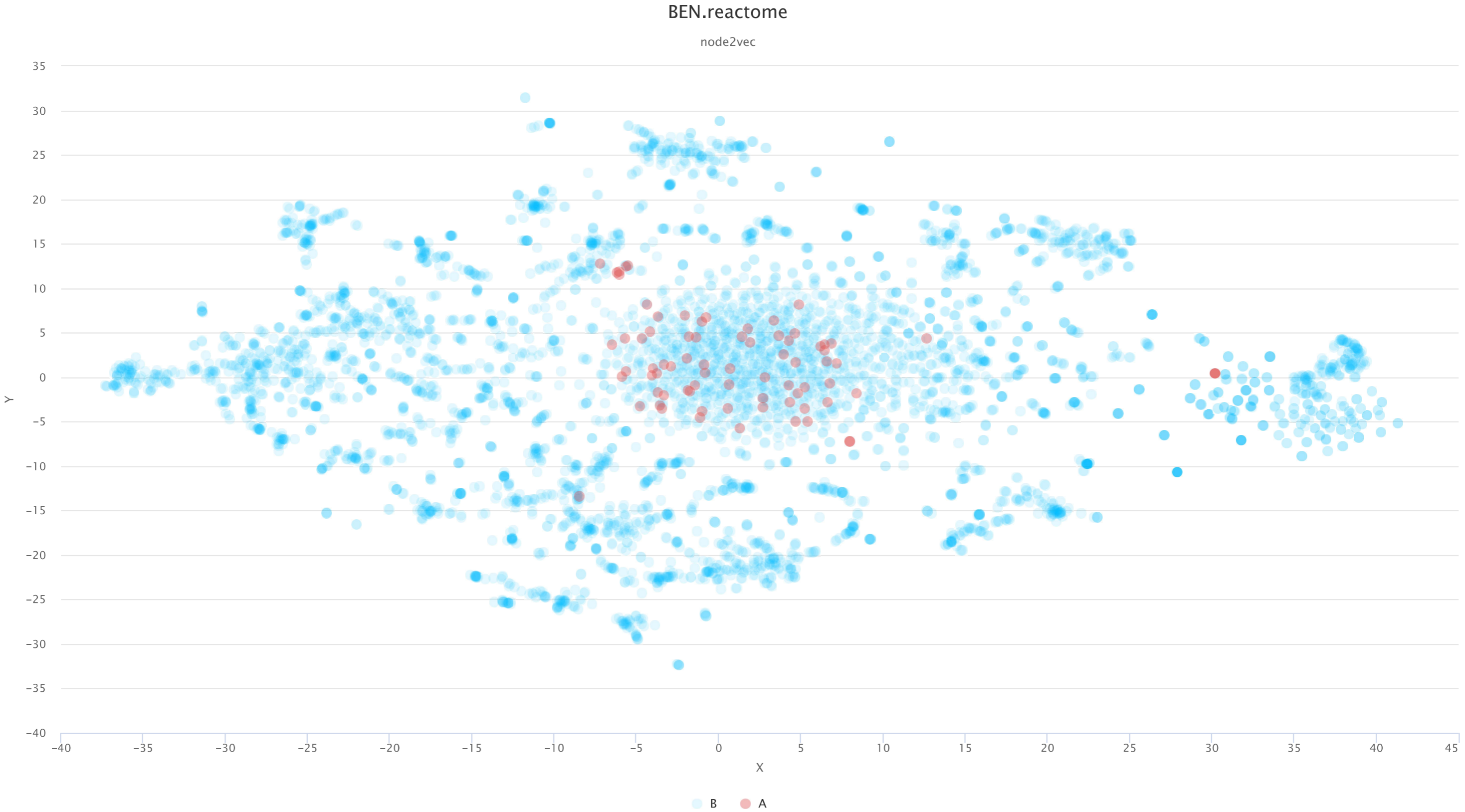

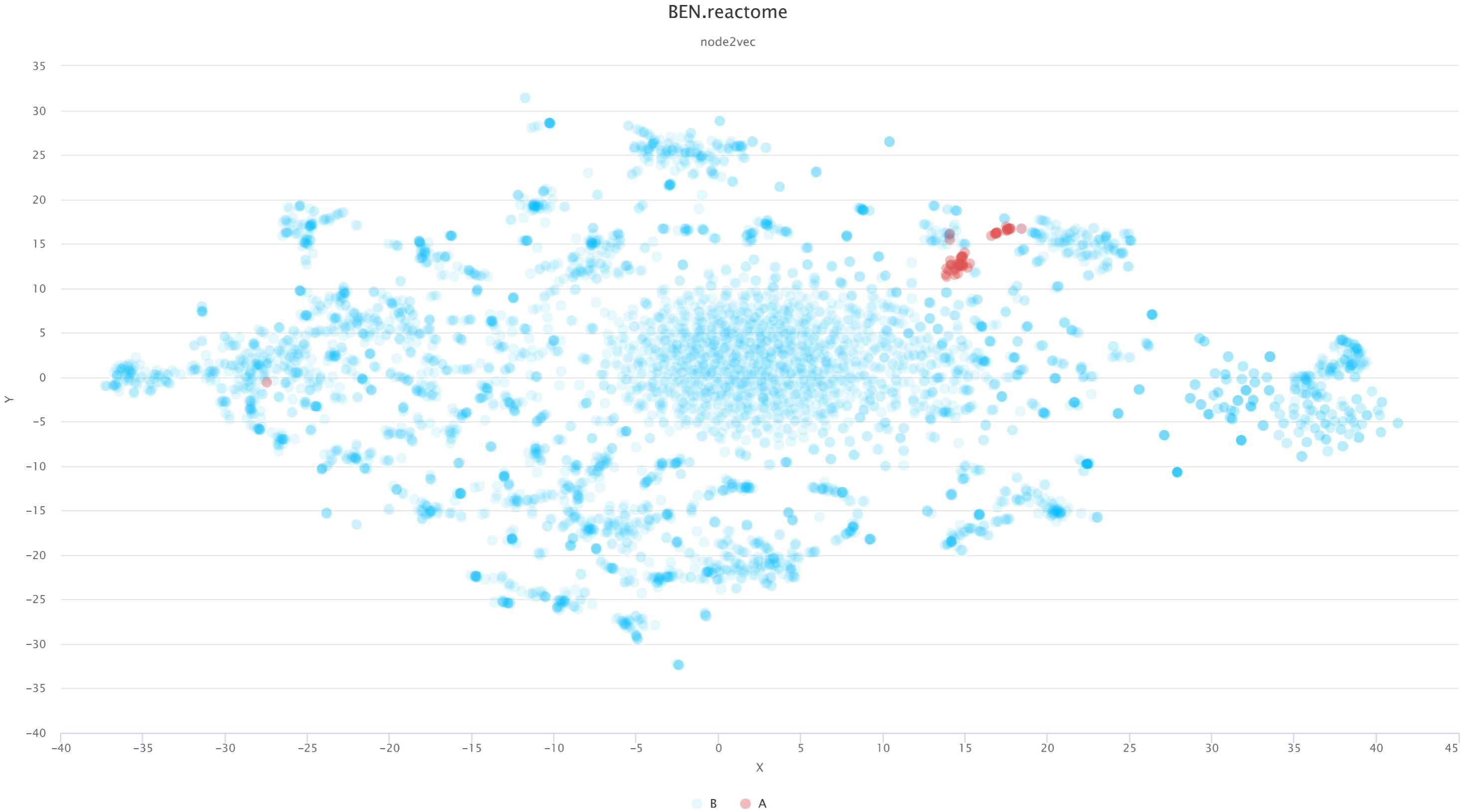

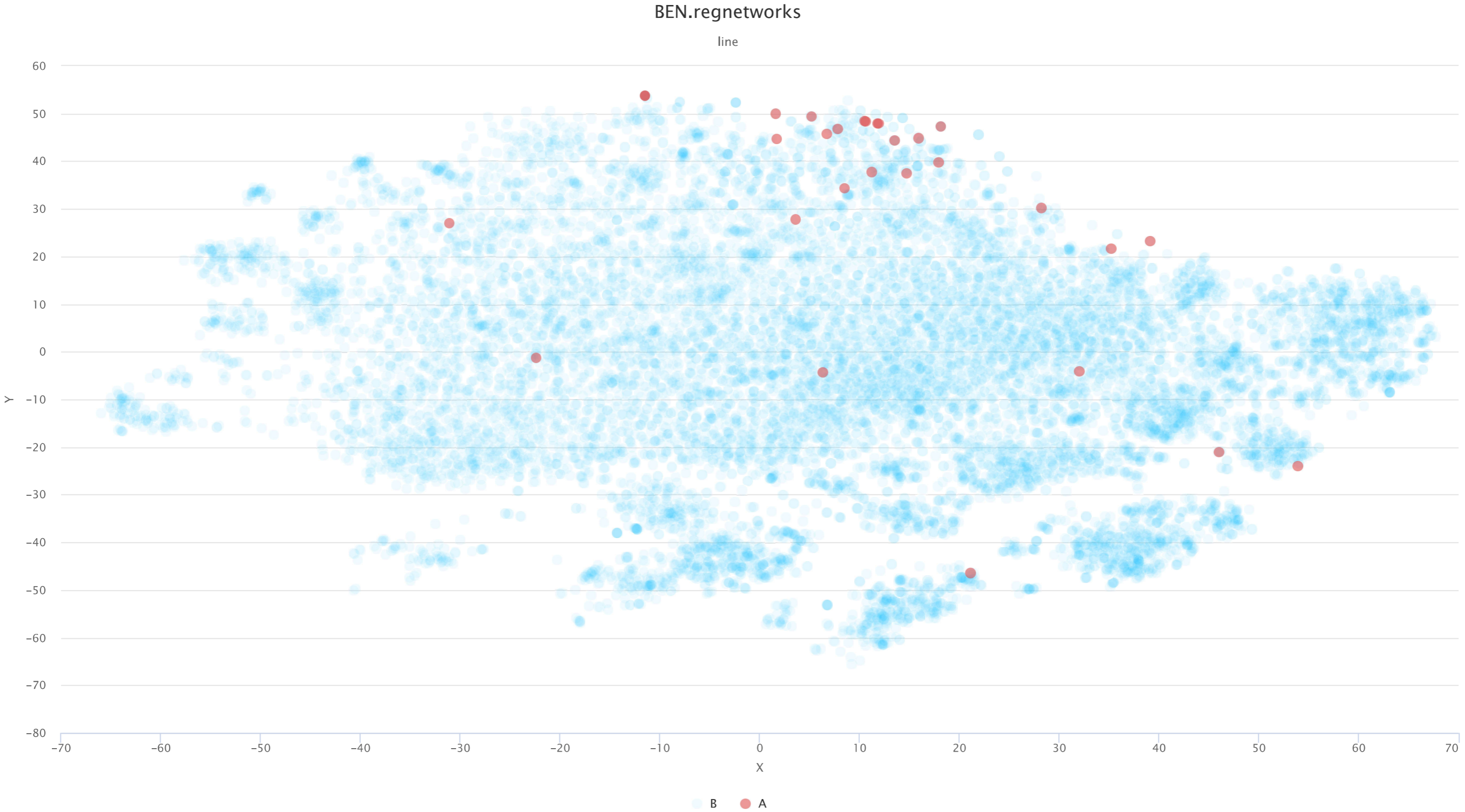

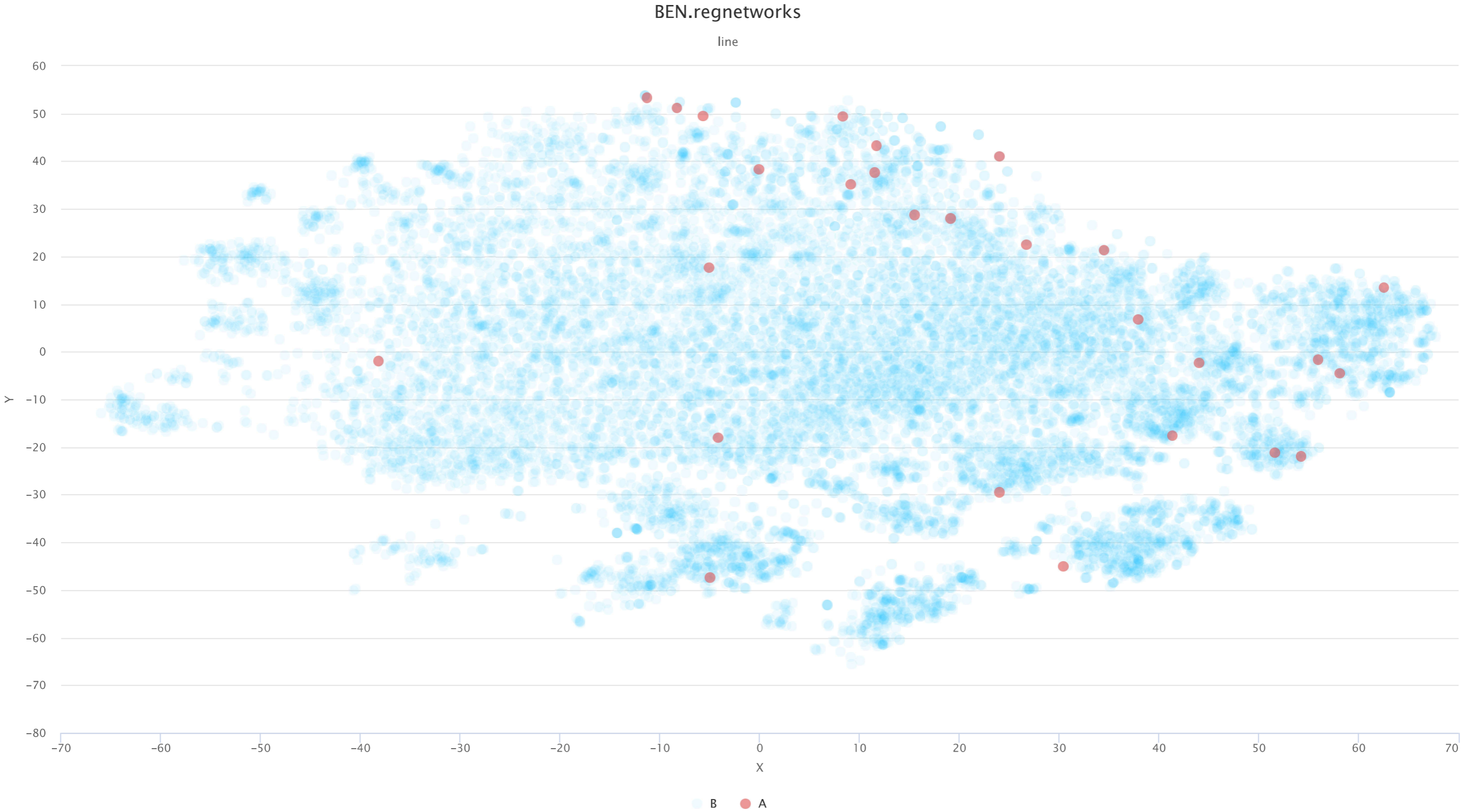

Supplement: baab033_Supp [file baab033_supp.zip › Supporting Material 1.pdf]
